# Left ventricular noncompaction and orthodromic atrioventricular tachycardia observed in a patient with neurofibromatosis type 1

**DOI:** 10.1093/omcr/omz021

**Published:** 2019-03-29

**Authors:** Martin Ibarrola, Andrés Ricardo Pérez-Riera, Mario D González

**Affiliations:** 1 Centro Cardiovascular BV, Bella Vista, Buenos Aires, Argentina; 2 Design of Studies and Scientific Writing Laboratory in the ABC School of Medicine, Santo André, São Paulo, Brazil; 3 Electrophysiology Program, Penn State University Heart and Vascular Institute, Penn State University College of Medicine, The Milton S. Hershey Medical Center, Hershey, PA, USA

## Abstract

Isolated left ventricular noncompaction (LVNC) was described for the first time in 1984. It is a rare congenital disease, characterized by prominent trabecular meshwork pattern and deep intertrabecular recesses, communicated with the left ventricular chamber. Clinical presentation varies from asymptomatic patients, to those developing supraventricular and ventricular arrhythmias, thromboembolism, heart failure and sudden cardiac death. We present an unusual case, where the patient with Neurofibromatosis type 1 presented with a wide complex orthodromic atrioventricular reentrant tachycardia (AVRT) and a diagnosis of left posterior paraseptal accessory pathway in association with LVNC.

## INTRODUCTION

### Case report

A 21-year-old previously asymptomatic Caucasian man, presented to the emergency department with symptomatic wide complex sustained tachycardia that required cardioversion for termination. His personal history was positive only for the use of recreational drugs (cannabis, synthetic drugs, LSD, ecstasy). He was diagnosed with mild form of genetic neurofibromatosis type 1 (NF1), Von Recklinghausen’s disease at 3 years old. His grandfather died from left atrial myxoma and heart failure at 74 years old.

**Figure 1:**
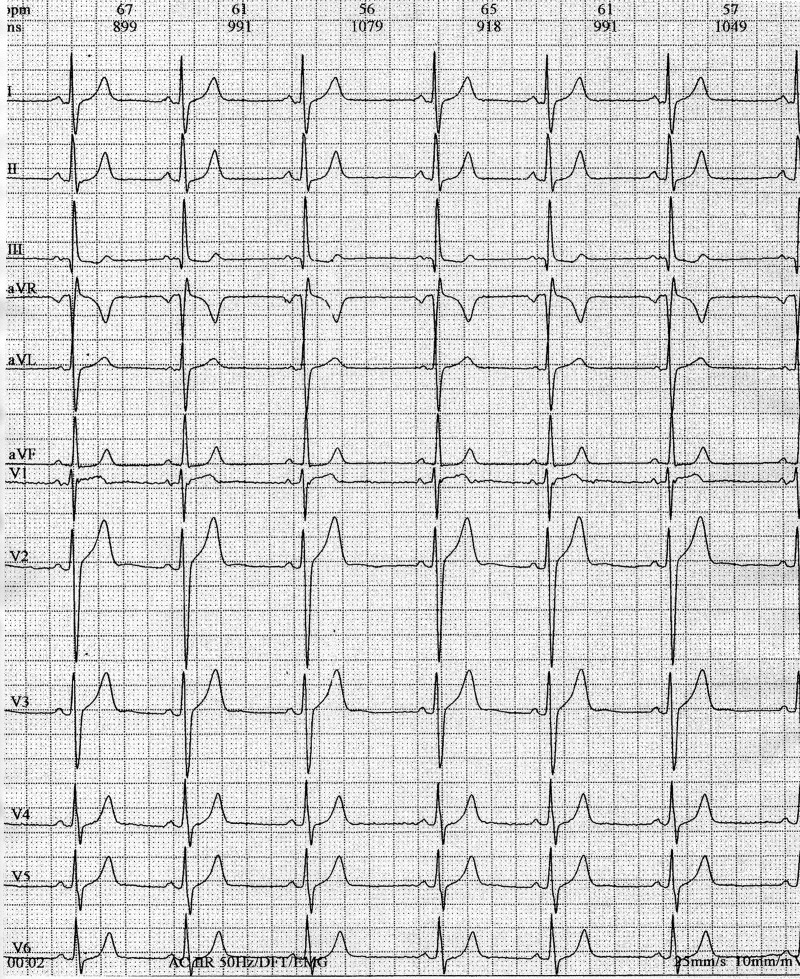
ECG: Sinus rhythm of 62 beats per minute. QRS axis 88°; PR interval 120 ms, QRS 98 ms, no abnormal findings.

Physical examination: Cardiovascular examination results and blood pressure were normal. Centripetal obesity. The body mass index was: 32. Had a six cafe-au-lait spots, measuring between to 5 to 12 mm. Two Lisch nodules were found in the eyes, and the head size was larger than average. Electrocardiogram was performed (Fig. 1).

**Figure 2:**
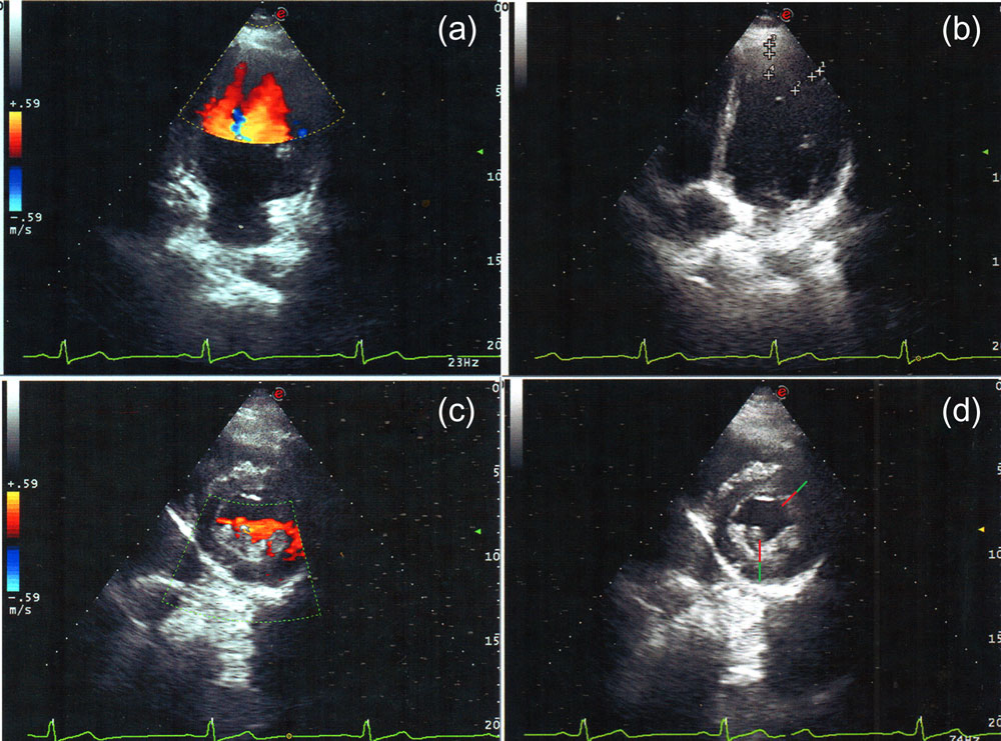
Echocardiogram: (**a**) Apical 4-chamber view, in color Doppler imaging where mild left ventricular dilatation is observed, noncompacted myocardium with trabeculations in apical wall; (**b**) Apical view where noncompacted myocardium (in red) and compacted myocardium ratio is observed (in green) ratio is >2; (**c**) Short axis left parasternal view; in color Doppler imaging multiple trabeculations and recesses observed in the lateral and inferior region, characteristic of noncompacted myocardium; (**d**) short axis left parasternal view the noncompacted myocardium (in red) and compacted myocardium (in green) ratio is >2.

Echocardiogram showed mild left ventricular (LV) dilatation, apical, lateral, and inferior hypokinesia, multiple and prominent myocardial trabeculations that communicate with the chamber in the same segments, and noncompacted/-compacted myocardium ratio of >2.5. (Fig. 2 and Video 1 shows the LVNC)

**Figure 3:**
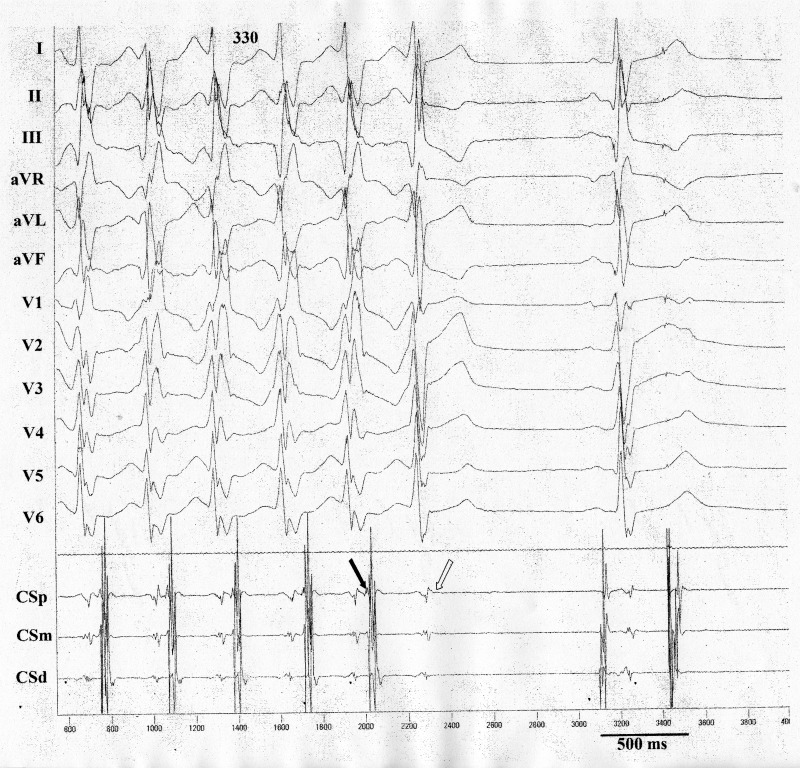
Electrophysiology study: Orthodromic atrioventricular reentrant tachycardia, using concealed accessory pathway located in the left posterior paraseptal region. The cycle length was 330 ms and the QRS complexes presented right bundle branch pattern. Earlier retrograde atrial activation was observed in the proximal coronary sinus (black arrow). During radiofrequency application, retrograde block was observed in the accessory pathway (absence of atrial activation: white arrow) with end of the event. After the pause, sinus beat is recorded with mild conduction delay in the right bundle branch.

In the electrophysiologyical study, orthodromic atrioventricular (AV) reentrant tachycardia was induced using as the retrograde limb, a concealed left posterior paraseptal accessory pathway located at 5 o’clock position in mitral valve annulus as observed from the left anterior oblique projection. Anterograde conduction over the AV node was associated with the rate- dependent block in the right bundle branch. Radiofrequency catheter ablation eliminated the accessory pathway conduction but terminated the tachycardia (Fig. 3).

## DISCUSSION

NF1 is usually diagnosed based on the presence of characteristic signs and symptoms. Specifically, physicians consider two or more of the following features to diagnose NF1. For the diagnosis six cafe-au-lait spots measuring >5 mm in all children and >15 mm in adults. Other signs and symptoms may include the following: (1) high blood pressure (potentially from renal artery stenosis or pheochromocytoma); (2) bone abnormalities; (3) optic nerve tumors; (4) Lisch nodules; (5) learning disabilities, attention deficit hyperactivity disorder, and autism spectrum disorder; (6) larger than average head size; (7) and short stature. Our patient has a minor expression of NF1, as confirmed by a genetic diagnostic test [1].

The frequency of congenital heart defects ranges from 0.4% to 6.4% in published series of NF1 patients [2]. Is unclear whether cardiovascular abnormalities are more common. NF1 associated with hypertrophic cardiomyopathy are also described [3]. The involvement of neurofibromin in cardiac development is strongly supported by Nf1 ‘knockout’ mouse models. Homozygous Nf1 mutant embryos succumb before Day 14 of gestation with double outlet right ventricle and associated abnormalities of cardiac outflow tract formation, endocardial cushion development, and myocardial structure [4].

LVNC associated with congenital heart disease was reported for the first time in 1926 [5]. Only in 1984, it was described as an echocardiographic finding with no other heart disease, isolated LVNC [6]. It is a heterogeneous and rare cardiomyopathy, which could be genetic familial mutation or sporadic, and its incidence in adults has been estimated as 0.05% [7]. LVNC is anatomically characterized by the presence of numerous prominent ventricular trabeculations and deep intertrabecular recesses; they are communicating with the ventricular chamber, caused by an alteration in cardiac embryogenesis. The most severe forms are observed mainly in children, due to multiple mutations or TTN mutation. The less severe ones are sporadic and more frequent in adults [8]. Its classification has no consensus yet. Early diagnosis and proper treatment for these patients is crucial because high morbidity and mortality may cause early heart failure, leading to potentially life-threatening supraventricular and ventricular arrhythmias and systemic embolisms [9].

The echocardiographic criteria of LVNC include: (1) the presence of ≥4 prominent trabeculations and deep intertrabecular recesses; (2) appearance of blood flow from the ventricular chamber to intertrabecular recesses that can be visualized using color Doppler imaging; (3) segments of noncompacted ventricle mainly involving the LV apex, inferior, and lateral wall and typically show a two -layer structure with telesystolic ratio of >2 between the noncompacted subendocardial layer and the compacted subepicardial one; and (4) absence of coexisting cardiac anomalies. The most widely used and accepted classification is proposed by Jenni *et al.*, who defined LVNC as a two-layer structure, with a thin layer, normally compacted (C) and a thicker, noncompacted (NC) (with an NC/C ratio >2), which were excessively prominent trabeculae and deep intertrabecular recesses measured at the end of systole in the short -axis parasternal projections. In hearts with prominent myocardial trabeculations due to other causes, the thickness ratio between trabeculated myocardium and normal ones does not reach >2. Moreover, trabeculated regions associated with LVNC tend to be segmentary instead of diffuse, because it occurs in the left ventricular hypertrophy [10, 11].

Electrophysiological findings include supraventricular tachycardia (SVT), ventricular pre-excitation of the Wolff-Parkinson-White syndrome, particularly in the pediatric population (present in up to 7% in this age group) [12], but rarely observed in adults. SVT as the first manifestation of LVNC is rarely observed in adults [13].

Herein, the patient characteristics associated with NF1 were confirmed by genetic testing. The patient also had LVNC, resulting in a wide complex orthodromic AV reentrant tachycardia due to aberrant conduction in the right bundle branch block pattern.

## CONCLUSION

This is a case of NF1 associated with LVNC. This association has not yet been reported previously and may only be casual. Therefore, prospective studies should be conducted to validate this association. In addition all patients with supraventricular tachycardia should be initially evaluated, to rule out structural heart disease, and as in the present case, a rare condition like LVNC should be carefully considered and closely followed up due to the potential for other complications related to cardiomyopathy.

## Supplementary Material

Supplementary Data

## References

[1] Smith A, Araoz PA, Kirsch J Coronary arterial aneurysms in neurofibromatosis 1: case report and review of the literature. J Thorac Imaging 2009;24:129–31. 19465837 10.1097/RTI.0b013e31819b684b

[2] Brannan CI, Perkins AS, Vogel KS, Ratner N, Nordlund ML, Reid SW, et al Targeted disruption of the neurofibromatosis type-1 gene leads to developmental abnormalities in heart various neural crest-derived tissues. Genes Dev 1994;8:1019–29. 7926784 10.1101/gad.8.9.1019

[3] Lin AE, Birch PH, Korf BR, Tenconi R, Niimura M, Poyhonen M, et al Cardiovascular malformations and other cardiovascular abnormalities in neurofibromatosis 1. Am J Med Genet 2000;95:108–17. 11078559 10.1002/1096-8628(20001113)95:2<108::aid-ajmg4>3.0.co;2-0

[4] Kizawa M, Nakagama Y, Shindo T, Ogawa S, Inuzuka R Identification of a novel titin variant underlying myocardial involvement in neurofibromatosis type 1. Can J Cardiol 2018;34:1369.e5–1369.e7. 10.1016/j.cjca.2018.07.473. 30269836

[5] Grant RT An unusual anomaly of the coronary vessels in the malformed heart of a child. Heart 1926;13:273–83.

[6] Engberding R, Bender F Identification of a rare congenital anomaly of the myocardium by two-dimensional echocardiography: persistence of isolated myocardial sinusoids. Am J Cardiol 1984;53:1733–4. 6731322 10.1016/0002-9149(84)90618-0

[7] van Waning JI, Caliskan K, Hoedemaekers YM, van Spaendonck-Zwarts KY, Baas AF, Boekholdt SM, et al Genetics, clinical features, and long-term outcome of noncompaction cardiomyopathy. J Am Coll Cardiol 2018;71:711–22. 29447731 10.1016/j.jacc.2017.12.019

[8] Maron BJ, Towbin JA, Thiene G, Antzelevitch C, Corrado D, Arnett D, et al Contemporary definitions and classification of the cardiomyopathies: an American heart association scientific statement from the council on clinical cardiology, heart failure and transplantation committee; quality of care and outcomes research and functional genomics and translational biology interdisciplinary working groups; and council on epidemiology and prevention. Circulation 2006;113:1807–16. 16567565 10.1161/CIRCULATIONAHA.106.174287

[9] Elliott P, Andersson B, Arbustini E, Bilinska Z, Cecchi F, Charron P, et al Classification of the cardiomyopathies: a position statement from the European Society of Cardiology Working Group on Myocardial and Pericardial Diseases. Eur Heart J 2008;29:270–6. 17916581 10.1093/eurheartj/ehm342

[10] Jenni R, Oechslin E, Schneider J, Jost C, Kaufmann P Echocardiographic and pathoanatomical characteristics of isolated left ventricular non-compaction: a step towards classification as a distinct cardiomyopathy. Heart 2001;86:666–71. 11711464 10.1136/heart.86.6.666 PMC1730012

[11] Ritter M, Oechslin E, Sütsch G, Attenhofer C, Schneider J, Jenni R Isolated noncompaction of the myocardium in adults. Mayo Clin Proc 1997;72:26–31. 9005281 10.4065/72.1.26

[12] Steffel J, Kobza R, Namdar M, Wolber T, Brunckhorst C, Lüscher TF, et al Electrophysiological findings in patients with isolated left ventricular non-compaction. Europace 2009;11:1193–1200. 19589795 10.1093/europace/eup187

[13] Lofiego C, Biagini E, Pasquale F, Ferlito M, Rocchi G, Perugini E, et al Wide spectrum of presentation and variable outcomes of isolated left ventricular noncompaction. Heart 2007;93:65–71. 16644854 10.1136/hrt.2006.088229 PMC1861346

